# Supplementary material for: Diffusive tail anchorage determines velocity and force produced by kinesin-14 between crosslinked microtubules
Source: Nat Commun. 2018 Jun 7;9:2214. doi: 10.1038/s41467-018-04656-0 (PMC5992172; doi:10.1038/s41467-018-04656-0)
Supplement: Supplementary file 1 — Supplementary Information [file 41467_2018_4656_MOESM1_ESM.pdf]

Supplementary Information

Lüdecke et al., **Diffusive tail anchorage determines velocity and force produced by kinesin-14 between crosslinked microtubules**

Supplementary Figures

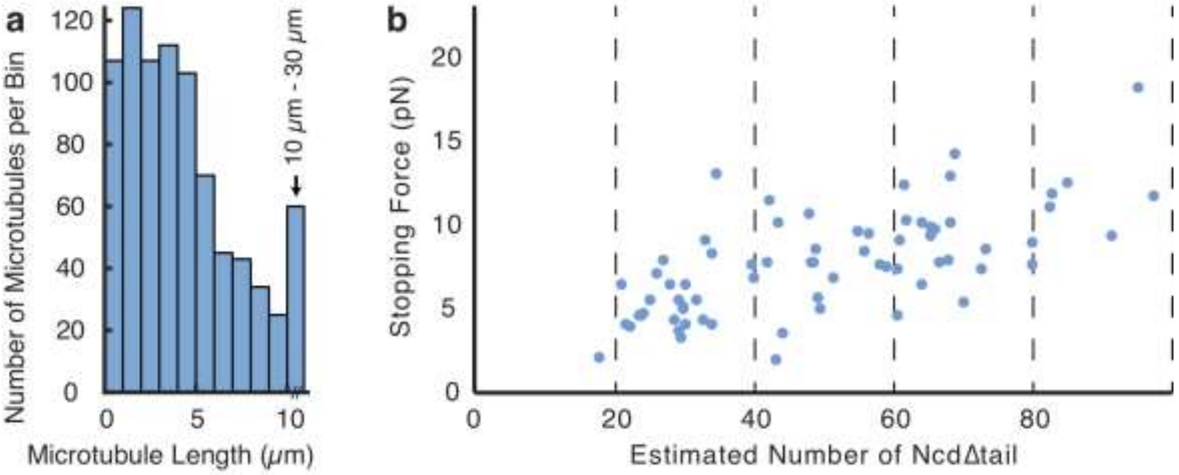

Supplementary Figure 1. (a) Number of microtubules in each bin in Fig. 1c. (b) individual measurements presented in Fig. 1f boxplots.

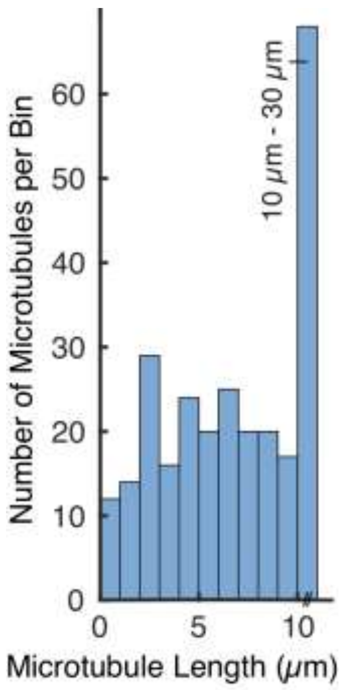

Supplementary Figure 2. Number of microtubules in each bin in Fig. 2c.

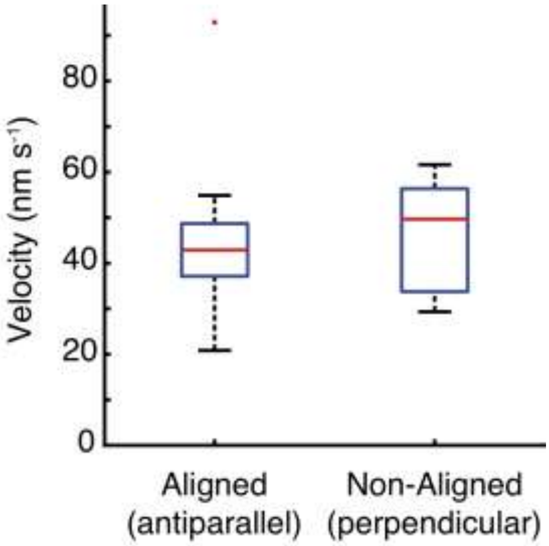

Supplementary Figure 3. Sliding velocity of microtubules propelled by kinesin-5 in aligned (antiparallel, 42 ± 10, mean ± SD, *N* = 35 microtubules) and non-aligned (perpendicular, 46 ± 11, mean ± SD, *N* = 12) geometry. For the boxplot elements description see Methods.

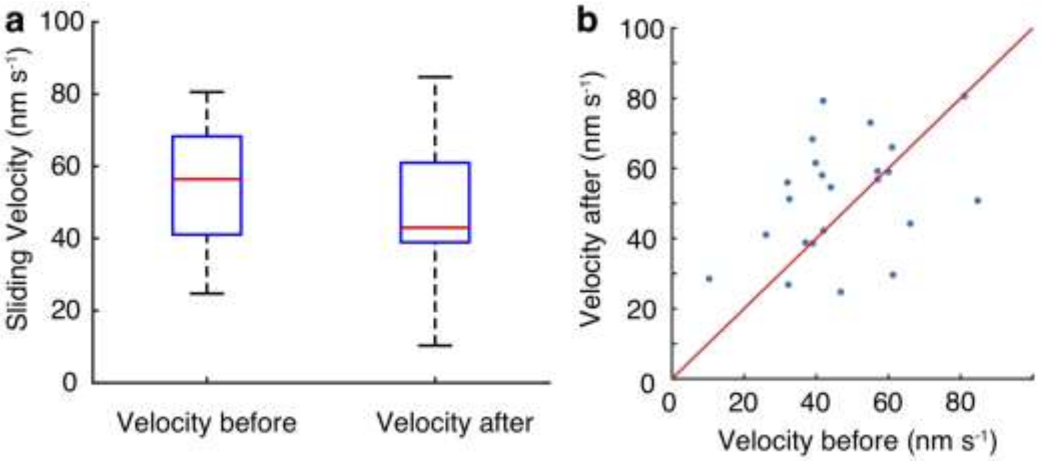

Supplementary Figure 4. Transport velocities (without external load) before (57 ± 23, mean ± SD) or after (50 ± 20, mean ± SD) the force-velocity measurements (*N* = 26). For the boxplot elements description see Methods. The red line marks equal velocities before and after force-velocity measurement.

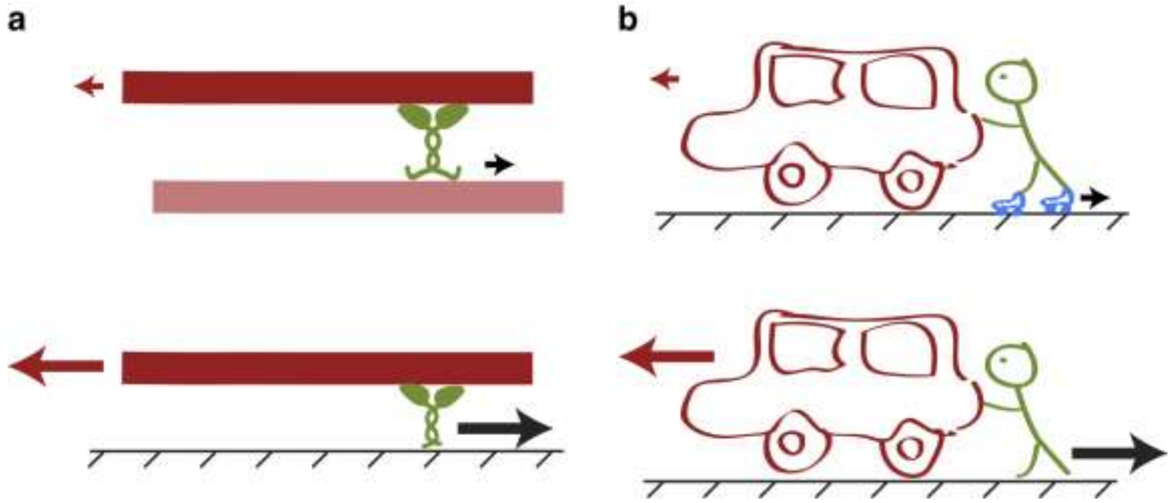

Supplementary Figure 5. Anchoring of the tail domain determines the force generated by kinesin-14 between two microtubules. (**a**) The force generated by the motors, transmitted between the microtubules (red arrows), is determined by the friction between the tail and microtubule (black arrows). Diffusively anchored motors (upper panel) generate lower forces and velocities than statically anchored motors (lower panel) because the energy, that the motor takes up - and could translate into forward motion of the microtubule dissipates during the backwards-slipping of the tail-domain. Static anchoring prevents slippage, thus allowing the motor to generate the maximum force, while slippery anchoring of the kinesin-14-tail limits the generated forces and velocities. Although non-processive kinesin-14 motors operate in ensembles, a simplified one-motor drawing is used to enhance the clarity of the argument. (**b**) A macroscopic analogy of this situation is a person pushing a car. When on roller-skates (upper panel), the person generates lower forces and velocities, due to the slipping of the roller-skates (red and black arrows represent the driving- and the frictional- forces respectively). Wearing shoes (as opposed to roller skates) provides static friction sufficiently high to prevent slippage, thus allowing the person, to generate the maximum force, while slippage of the roller skates limits the generated forces and velocities, with the maximal generated force being equal in magnitude to the frictional resistance force that the roller skates will provide.
