# Supplementary material for: Diffusive tail anchorage determines velocity and force produced by kinesin-14 between crosslinked microtubules
Source: Nat Commun. 2018 Jun 7;9:2214. doi: 10.1038/s41467-018-04656-0 (PMC5992172; doi:10.1038/s41467-018-04656-0)
Supplement: Supplementary file 3 — Description of Additional Supplementary Files [file 41467_2018_4656_MOESM3_ESM.pdf]

Description of Additional Supplementary Files

File Name: Supplementary Movie 1
Description: Exemplary movie showing fluorescently labeled, gliding microtubules propelled by statically anchored NcdΔtail. See Fig. 1b for the quantification of the gliding velocities.

File Name: Supplementary Movie 2
Description: Exemplary movie showing fluorescently labeled, sliding microtubules propelled by GFP-flNcd. See Fig. 2b for the quantification of the velocities of the aligned, sliding microtubules. Please note the two non-aligned microtubules sliding perpendicularly to the surface immobilized microtubules, as described in Fig. 3a,b.

File Name: Supplementary Movie 3
Description: Exemplary movie showing the buckling of a fluorescently labeled microtubule, propelled by GFP-flNcd, as described in Fig. 2h,i.
